# Binding Parameters of [^11^C]MPC-6827, a Microtubule-Imaging PET Radiopharmaceutical in Rodents

**DOI:** 10.3390/ph16040495

**Published:** 2023-03-27

**Authors:** Avinash H. Bansode, Bhuvanachandra Bhoopal, Krishna Kumar Gollapelli, Naresh Damuka, Ivan Krizan, Mack Miller, Suzanne Craft, Akiva Mintz, Kiran Kumar Solingapuram Sai

**Affiliations:** 1 Department of Radiology, Wake Forest School of Medicine, Winston Salem, NC 27157, USA; 2 Department of Gerontology, Wake Forest School of Medicine, Winston Salem, NC 27157, USA; 3 Department of Radiology, Columbia Medical Center, New York, NY 10032, USA

**Keywords:** PET imaging, microtubules, radiochemistry, metabolism, stability, translational molecular imaging

## Abstract

Impairment and/or destabilization of neuronal microtubules (MTs) resulting from hyper-phosphorylation of the tau proteins is implicated in many pathologies, including Alzheimer’s disease (AD), Parkinson’s disease and other neurological disorders. Increasing scientific evidence indicates that MT-stabilizing agents protect against the deleterious effects of neurodegeneration in treating AD. To quantify these protective benefits, we developed the first brain-penetrant PET radiopharmaceutical, [^11^C]MPC-6827, for *in vivo* quantification of MTs in rodent and nonhuman primate models of AD. Mechanistic insights revealed from recently reported studies confirm the radiopharmaceutical’s high selectivity for destabilized MTs. To further translate it to clinical settings, its metabolic stability and pharmacokinetic parameters must be determined. Here, we report *in vivo* plasma and brain metabolism studies establishing the radiopharmaceutical-binding constants of [^11^C]MPC-6827. Binding constants were extrapolated from autoradiography experiments; pretreatment with a nonradioactive MPC-6827 decreased the brain uptake >70%. It exhibited ideal binding characteristics (typical of a CNS radiopharmaceutical) including LogP (2.9), *K*_d_ (15.59 nM), and *B*_max_ (11.86 fmol/mg). Most important, [^11^C]MPC-6827 showed high serum and metabolic stability (>95%) in rat plasma and brain samples.

## 1. Introduction

Alzheimer’s disease (AD) is recognized by the formation of extracellular amyloid plaques and intracellular hyperphosphorylated tau, a microtubule-associated protein (MAP) [1,2]. Changes in tau protein in axonal region (axonal MAP) are associated with AD and other related dementia disorders [3,4,5,6,7,8,9,10,11,12,13]. Tau normally functions as a bridge, ensuring that MTs in axons run straight and parallel to one another. In the pathological changes implicated in AD and related dementias, tau detaches from MTs and accumulates in the neuronal cell body [14,15]. The flow of information in the neurons is affected due to withering of axons caused by the disruption of cytoskeleton [16]. Additionally, MT dynamics contribute to changes in neuronal polarities, altering MAP distribution and resulting in neurodegeneration events [17]. MT stability is disrupted early in AD and other related dementias, primarily when abnormal Aβ and tau modifications sequester MAPs. Taken together, these findings suggest that MT dysfunction connects Aβ/tau-based degenerative events to the hallmark pathologies of AD. Therefore, quantifying MT dynamics is critical in understanding early neurodegenerative cascade of AD [17,18].

Neuroimaging has revolutionized the identification and quantification of molecular changes during neurodegeneration and currently available Aβ and tau PET can characterize disease lesions [19,20,21]. We hypothesized that MT-based PET can target early molecular events contributing to AD pathophysiology, which is considered a key gap in the understanding of MT structural changes inherent to AD pathophysiology. 

MPC-6827 is a high-affinity MT agent (~1.5 nM) that demonstrated suppression of tumor growth in a variety of cancer animal models. It has been proven safe to use in human subjects and ideal pharmacokinetics and has undergone multiple clinical trials for treatment of glioblastoma and other advanced cancers [22,23]. MPC-6827 crosses the blood–brain barrier in mice, rats and dogs and is distributed rapidly in brain with approximately 14–30 times brain to plasma ratios [24]. O-desmethyl-MPC was the major metabolite of MPC-6827 in humans [23]. Therefore, PET radiolabeling of MPC-6827 at this site probably lead to non-radioactive metabolites and may not interfere with the binding outcome [23,25]. Although clinical outcome suggests that the drug as single agent or combination may have limited success in cancer therapy [26], high brain penetration, lack of multiple drug resistance and established safety profile in humans are the merits of [^11^C]MPC-6827 as a potential CNS PET imaging agent.

To study MT destabilization and its progression in all dementia disorders including AD, we developed the first brain-penetrating PET radiopharmaceutical, [^11^C]MPC-6827 [27], and evaluated its imaging efficacy in rodents and nonhuman primate models of AD [27,28,29,30]. Additionally, our new *in vitro*, *in vivo* and *ex vivo* mechanistic studies showed that [^11^C]MPC-6827 uptake is higher to destabilized MTs than stabilized MTs and that the uptake increases with brain Aβ/tau burden [31]. Postmortem studies on patients with confirmed AD showed higher radiopharmaceutical uptake than the cognitively normal age-matched controls [31]. Given the extensive work published from our group on [^11^C]MPC-6827, the next steps would be to quantify its binding and metabolic characteristics in target (brain) and plasma—these data will strengthen its potential as a CNS PET radiopharmaceutical. Additionally, to further translate the radiopharmaceutical’s promise to clinical settings, we must first characterize its binding and pharmacokinetic properties, including binding constants, plasma and brain metabolism, and stability. Here, we report *in vitro*, *in vivo* and *ex vivo* findings on [^11^C]MPC-6827 binding and metabolism in rodents.

## 2. Materials and Methods

### 2.1. Radiochemistry

[^11^C]MPC-6827 was produced based on our previously published methods with slight modifications to better shorten reaction times [27]. Briefly, Wake Forest PET Center GE-PETtrace-800 cyclotron generated [^11^C]CO_2_ was converted to [^11^C]methane through nickel catalyst at 360 °C with GE-FXC radiochemistry module. [^11^C]methyl iodide (MeI) was formed from the reaction of [^11^C]methane with gaseous iodine (I_2_) at 760 °C. The desmethyl precursor (0.8–1 mg) and 5 µL 5 N NaOH solution were heated for 3 min at 80°C in a closed reaction glass vial, post [^11^C]MeI delivery. Then, 0.7 mL of HPLC mobile phase was used to quench the reaction mixture, which was then transferred onto a C18 Phenomenex ODS (250 × 10 mm, 10 μ) semi-preparative HPLC column for purification of [^11^C]MPC-6827. HPLC mobile phase solution consisted of acetonitrile and 0.1 M aqueous ammonium formate solution (4:6), pH 6.0–6.5, UV λ_max_ of 254 nm, and a 5.0 mL/min flow rate. The final radiopharmaceutical, [^11^C]MPC-6827 with a retention time (Rt) of 8–9 min, was collected in a round bottom flask preloaded with sterile water (~50 mL), and then allowed to pass through a C18 SepPak cartridge (WAT036800, Waters) to retain the desired product. [^11^C]MPC-6827 was eluted from the radioactive cartridge with ethanol (1.0 mL) and aqueous saline solution to a final concentration of 10% ethanol in saline. The product was directly collected into a clean sterile final product vial (FPV) through a 0.22 μm Millipore sterile filter (R1AB86553, Millipore Corp.) for QC analyses and further biological studies. The pH of [^11^C]MPC-6827 was determined using pH paper, and the final purity was determined using an analytical quality control (QC) HPLC system (C18 Phenomenex HPLC column [250 × 4.6 mm, 5 μ]) and UV λ_max_ of 254 nm and a flow rate of 1 mL/min. The QC mobile phase consisted of acetonitrile and 0.1 M aqueous ammonium formate solution (6:4) with a pH 6.0–6.5. [^11^C]MPC-6827 was validated by performing a routine co-injection with the non-radioactive standard MPC-6827. Quality assessments including specific activity, radiochemical purities and associated mass were determined at end of synthesis of the final product, [^11^C]MPC-6827 absorption and standard calibration peak curves (UV λ_max_ = 254 nm). 

### 2.2. Lipophilicity (LogP)

The sample (2 mL) containing 3.7 MBq [^11^C]MPC-6827 in PBS buffer, pH 7.4 was mixed with 2 mL 1-octanol, vortexed for 10 min, followed by centrifugation at 5000× *g* for 5 min. The top layer (organic) and the bottom (aqueous) layers were separated and transferred into different tubes. All the tubes, including the control tube, were γ-counted and % of radiopharmaceutical in each solvent was determined to calculate LogP. 

### 2.3. Serum Stability

[^11^C]MPC-6827 (∼37–40 MBq) was added to human serum to bring the final volume to 1 mL *ex vivo* followed by incubation at 37 °C. [^11^C]MPC-6827 and the radioactive serum mixture (∼50 μL) were injected into a QC-HPLC (analytical reverse phase Phenomenex HPLC column, 250 × 4.6 mm; 5 μ and UVmax = 254 nm; flow rate = 1.0 mL/min; mobile phase = 60% acetonitrile and 40% 0.1 M aqueous ammonium formate [pH 6.0–6.5] solution; Rt = 5–6 min) at 5 min, 30 min, 1 h, 1.5 h, 2 h, and 3 h after radiopharmaceutical synthesis. 

### 2.4. Binding Constants

For *in vitro* autoradiography experiments, mouse brain tissues were sectioned sagitally using a Leica cryostat at 20 µm thickness, mounted onto Superfrost™ Plus slides. Sections were stored at −80 °C until further use. Before incubation for 30 min at room temperature with [^11^C]MPC-6827, sections were kept for thawing for 10 min followed by rehydration in PBS, pH 7.4 for 5 min. Different concentrations of [^11^C]MPC-6827 were used (63.5, 31.75, 15.87, 7.9, 3.95, 1.97, 0.98, and 0.49 nM) in PBS for the experiment. Adjacent sections were incubated with the same concentration range of [^11^C]MPC-6827 containing 200 µM MPC-6827. Brain sections were washed in PBS (4 × 3 min) and briefly dipped in water. After drying, the sections were exposed to a BAS IP SR 2025 E imaging plate for 10 h. Typhoon Phosphor imaging system was used to scan the plate. Image analysis and quantification was carried out using Fiji (ImageJ, NIH). 

The homologous binding assay was carried out with [^11^C]MPC-6827 and unlabeled MPC-6827 as a blocking agent. Briefly, brain homogenate was prepared from normal male mice (*n* = 6, age 4–5 months) and stored as homogenate solutions (40 mg/mL). The homogenate solution was diluted in PBS buffer, pH 7.4 to a final concentration of 10 µg protein in 250 μL solution per sample. A 50 μL aliquot from a series of [^11^C]MPC-6827 solutions at increasing concentrations (2.5, 5, 10, 15, 20, 25, 40 and 60 nM) was added to each sample tube. Next, 50 μL aliquots of MPC-6827 standard solution (200 µM) and appropriate amounts of binding buffer were added to each sample to reach a final volume of 250 μL per sample. All the samples were incubated at 37 °C for 30 min, then transferred to a 96-well filter plate (PerkinElmer) and washed with PBS buffer (200 μL). The filters were dried at 55 °C for 10 min, harvested and γ-counted using the PerkinElmer γ-counter. Counts-per-minute (CPM) values from the γ counter were decay-corrected and converted to fmol/mg values. Specific binding data were fitted nonlinearly to estimate the *K_d_* and *B_max_* (maximum specific binding) values from Graphpad Prism v9.3.1.

### 2.5. Plasma Metabolite Assays

Having demonstrated its lipophilicity, serum stability and binding constants in vitro and *ex vivo*, the next studies focused on the metabolic stability of [^11^C]MPC-6827 in rat brain and plasma tissues *in vivo*. The percentage of [^11^C]MPC-6827 radioactivity in rat plasma (male Sprague-Dawley rats, *n* = 4 per time point) was determined by C18 HPLC system. Around 0.5 mL of blood samples was drawn from rats at 5, 15, 30, 60 and 90 min after radiopharmaceutical injection. Plasma (0.2 mL) was separated from blood samples via centrifugation, and then added to acetonitrile (0.3 mL), vortexed (~10 s) and centrifuged at 14,000 rpm (for 5 min). The resulting supernatant liquid (0.3 mL) was separated and diluted with water (0.3 mL), and the radioactivity was measured via a γ-counter before being injected into the same QC HPLC column conditions, equipped with a radioactivity detector. Radioactive metabolite and parent radiopharmaceutical samples were collected, gamma counted and corrected for background radioactivity to measure parent concentration/percentage in the plasma at various time points. A QC sample of [^11^C]MPC-6827 was injected at the beginning and end of the study to ensure that the parent retention time had not shifted during metabolite analysis. The metabolite fractions per time point were calculated by dividing the fractions corresponding to radioactivity counts of the parent radiopharmaceutical with total HPLC fraction counts.

### 2.6. Brain Metabolite Assays

The whole brain was harvested from male Sprague–Dawley rats (*n* = 4 per time point) to determine metabolite analysis. Excess blood was blotted; brain removed and carefully homogenized on ice, and radioactivity extracted by adding acetonitrile (1.2 mL) and homogenized again. An aliquot of this brain homogenate (1.0 mL) was centrifuged to pellet the debris. The clear supernatant (0.2 mL) was diluted with distilled water (0.2 mL) for HPLC injection as described above at the same time points: 5, 15, 30, 60 and 90 min after radiopharmaceutical administration. 

## 3. Results

**Figure 1:**
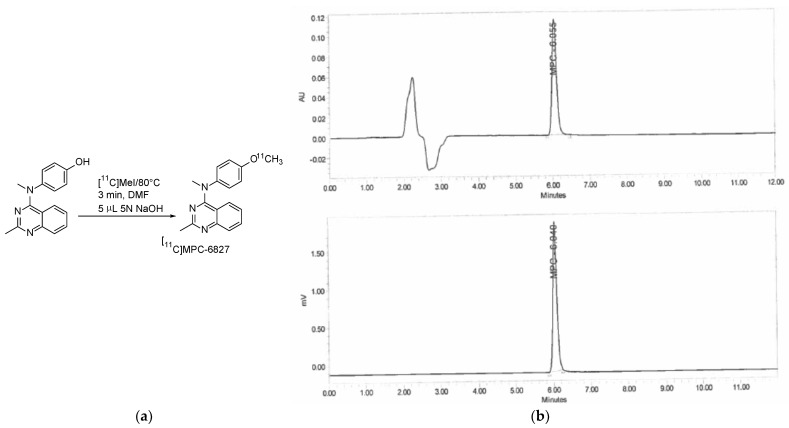
(**a**) Radiochemical scheme of [^11^C]MPC-6827 production; (**b**) associated QC-HPLC of a co-injection with MPC-6827, QC conditions: Isocratic HPLC with 40% acetonitrile and 60% 0.1 M aqueous ammonium formate solution (pH 6.0–6.5) at a UV wavelength of 254 nm and a flow rate of 1.0 mL/min [reverse-phase Prodigy ODS-3 (250 × 4.6 mm, 5 µm)].

High-quality [^11^C]MPC-6827 was produced [27], with a radiochemical yield of ~45% in >99% radiochemical purity and specific activity of >350 GBq/µmol, decay-corrected to the end of synthesis (*n* = 30). Test–retest property of [^11^C]MPC-6827 radiochemistry on all the specifications showed statistically significant correlation with r = 0.67 (*n* = 30 production runs). The final pH of [^11^C]MPC-6827 was 6.5–7.0. QC-HPLC analysis showed an average mass per batch production of 10–15 µg, and radiochemical purity was authenticated by co-injection with a nonradioactive MPC-6827, which demonstrated retention time similar to that of [^11^C]MPC-6827 (Figure 1).

A compound lipophilicity is usually indicated by LogP value [32]. The average percent recovery was ~98%. With a low molecular weight (315.8 g/mol), LogP of [^11^C]MPC-6827, i.e., octanol/water partition coefficient was ~2.9 ± 0.02 (*n* = 4). [^11^C]MPC-6827 efficiently crosses the blood–brain barrier, as previously reported in our PET imaging study [28]. 

Serum stability is important for a diagnostic imaging agent as it dictates the metabolic fate of the radiopharmaceutical in plasma and target tissue binding. The *ex vivo* serum stability in a human serum sample at different time-points was analyzed by QC HPLC analysis of aliquots extracted at different time points. At 3.0 h, >95% of the parent radioactive tracer remained intact, which indicates that [^11^C]MPC-6827 has high serum stability and is suitable for *in vivo* use. (Table 1), with a statistical significance of * *p* = 0.05.

**Figure 2:**
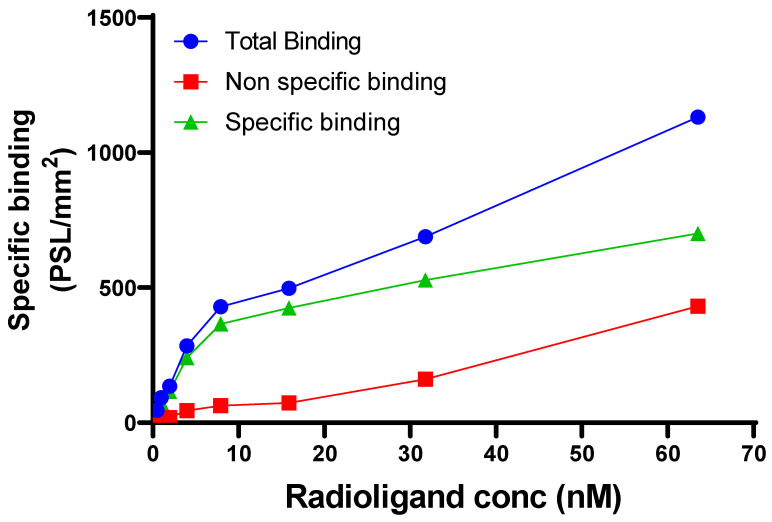
Binding assay of [^11^C]MPC-6827 using autoradiography studies.

Mouse brain sections were used for performing saturation-binding assay. Co-incubation with non-radioactive MPC-6827 (200 µM) reduced the total binding signal significantly (>70%). A linear increase in nonspecific binding was observed with tracer concentration. Saturation of total binding was observed at radiopharmaceutical concentrations above 20 nM. The *K*_d_ value for [^11^C]MPC-6827 is determined as 10.03 nM through autoradiography studies. (Figure 2)

**Figure 3:**
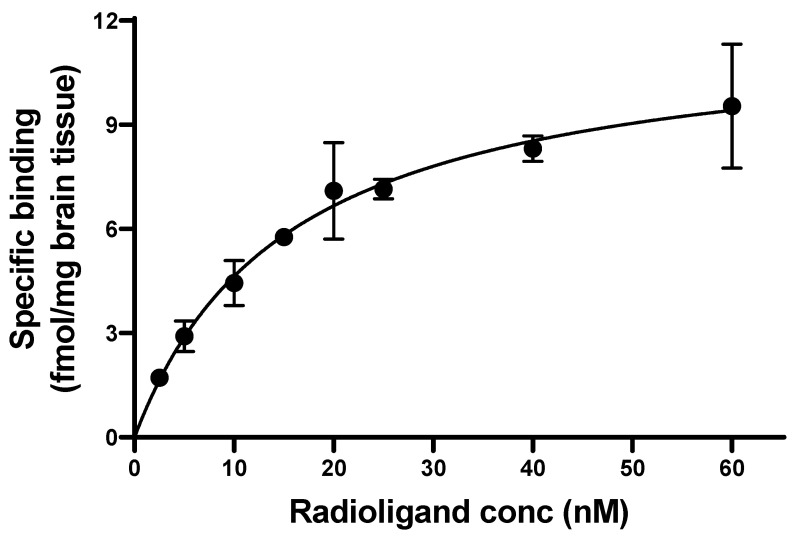
Binding assay of [^11^C]MPC-6827 in mouse brain homogenates (*n* = 6).

To determine specific [^11^C]MPC-6827 binding to mouse brain, a competitive binding assay was performed. The dissociation constant *K*_d_ was determined as 15.59 nM with *B*_max_ of 11.86 (fmol/mg), with a statistical significance of * *p* = 0.04 (Figure 3).

**Figure 4:**
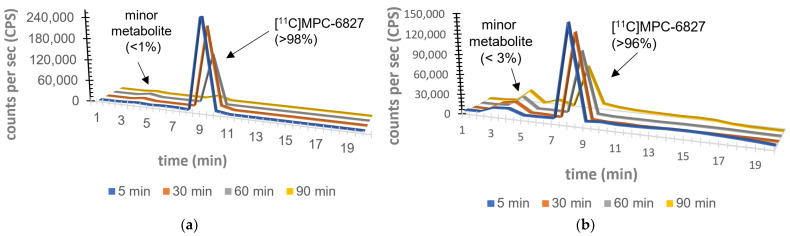
Metabolic assay of [^11^C]MPC-6827 in rat (**a**) plasma; (**b**) brain sample. (*n* = 4/time-point).

HPLC metabolite analysis [33] of the plasma and brain samples indicated that the percentage of the parent unmetabolized [^11^C]MPC-6827 was 98.5 ± 1.5% of total plasma radioactivity at 5 min; 97.7 ± 0.88% at 15 min; 97.1 ± 1.1% at 30 min; 95.7 ± 12.9% at 60 min; and 93.6 ± 2% at 90 min. With brain samples, the percentage of [^11^C]MPC-6827 was 99 ± 0.8% at 5 min; 98.2 ± 1.5% at 15 min; 97.1 ± 2.2% at 30 min; 95.4 ± 1.5% at 60 min; and 94.3 ± 2.0% at 90 min. HPLC analysis showed a minor peak with a retention time of 3–4 min in both plasma and brain samples. The extraction efficiency and column efficiency for all metabolite analyses were ~92 ± 1% and test–retest characteristics were statistically significant (*r* = 0.75). (Figure 4).

## 4. Discussion

PET imaging allows us to study biochemical, physiological and pharmacological functions at the cellular and molecular levels [34,35]. Inside the living organism, PET radiopharmaceuticals encounter various chemical effects including redox reactions, hydrolysis, decarboxylation and other conjugation processes. In order to assess PET radiopharmaceutical’s imaging efficacy to target regions, it is necessary to quantify the parent radiopharmaceutical’s fraction, binding and kinetics in both plasma and the target (here the brain tissue). 

Lipophilicity can be used to estimate how well the CNS-based PET radiopharmaceutical can penetrate the blood–brain barrier [36]. The common way to define lipophilicity is determining LogP, i.e., the partition coefficient of the compound between n-octonol and water. An ideal brain-penetrant radiopharmaceutical has a LogP of 2–3. [^11^C]MPC-6827 demonstrated a LogP of 2.9, making it possible to cross the blood–brain barrier. To confirm the stability of the radiopharmaceutical *ex vivo*, we applied QC-HPLC method to human serum sample and found that [^11^C]MPC-6827 was >95% stable in serum for at least 3 h post-injection; highly favorable for a [^11^C]-based PET isotope with a half-life of 20 min. 

Disassociation constant (*K*_d_) and binding specificity (*B*_max_) are two parameters used routinely to measure radiopharmaceutical affinity for the target tissue [37,38]. CNS-based radiopharmaceuticals with *K*_d_ and *B*_max_ values around 8–16 nM and <500 nM, respectively, showed decent to good target uptake with favorable binding and washing kinetics in neuro PET imaging. [^11^C]MPC-6827 showed a *K*_d_ of 15.59 nM and a *B*_max_ of 11.86 (fmol/mg) demonstrating that it is highly suitable for neuroimaging with a favorable pharmacokinetic profile.

As a CNS-based radiopharmaceutical, identification of radiometabolites of [^11^C]MPC-6827 is vital for translational PET imaging. Metabolic stability in plasma is crucial for the exact quantification of [^11^C]MPC-6827 that does not hinder the image analyses’ approximations for target and reference region in the brain. Determination of parent radiopharmaceutical in plasma at different time points will clarify the trajectory of [^11^C]MPC-6827 and provide important information on physiological, biochemical and pathological processes. The most widely used technique for the analysis of a radiopharmaceutical is HPLC, combined with radioactivity detectors. Radio-HPLC analyses demonstrated >95% parent radiochemical identity in both brain and plasma samples 90 min post-injection in male Sprague–Dawley rats. At 60 min and 90 min post-injection, radioactivity of plasma displayed a minor peak with a retention time of 3–4 min ([^11^C]MPC-6827 retention time is 5–6 min) on a C18 HPLC column. The percentage of this peak was ~2.8 ± 0.04% (at 60 min) and ~3.2% (at 90 min), while the parent radiopharmaceutical remained the major constituent (>97.5%). For the rat brain samples, similar percentages of radiometabolite with a retention time of 3–4 min were seen at 60 min (~1.6 ± 0.05%) and 90 min (~2.89 ± 0.11%). As the radiometabolite retention time in both brain and plasma samples was similar, it might be the same metabolite that crosses the blood–brain barrier. While an additional LC-MS analysis is needed to analyze its structure, based on the retention time on a C18 HPLC column, the (minor) metabolite may be slightly more lipophilic than the parent radiopharmaceutical [^11^C]MPC-6827.

This study demonstrated favorable binding and metabolic parameters of [^11^C]MPC-6827, following the routine protocols needed to test a CNS-based PET radiopharmaceutical. However, mass spectral analyses are needed to characterize the minor radiometabolite fraction. Next steps would be evaluating the imaging potential of [^11^C]MPC-6827 in non-human primates and humans using plasma metabolite assays, dosimetry and whole-body distribution.

## 5. Conclusions

In summary, [^11^C]MPC-6827, a high-affinity highly selective radiopharmaceutical for imaging MTs, was evaluated to determine its binding, pharmacokinetic stability and metabolic parameters. It exhibited ideal binding characteristics (typical of a CNS radiopharmaceutical) including LogP (2.9), *K*_d_ (15.59 nM), and *B*_max_ (11.86 fmol/mg). Most importantly, [^11^C]MPC-6827 showed high serum and metabolic stability (>95%) in rat plasma and brain samples. Metabolic analysis in these samples demonstrated only one minor radiometabolite, at <~3% with parent radiopharmaceutical >95%. Next steps include radiometabolite analysis and dosimetry studies in non-human primates. [^11^C]MPC-6827’s *in vitro*, *in vivo* and *ex vivo* binding characteristics demonstrate its high potential as a PET radiopharmaceutical for imaging MTs in humans, aiding in imaging applications for Alzheimer’s disease and related dementia and other therapeutic intervention strategies.

## Figures and Tables

**Table 1:** Stability of [^11^C]MPC-6827 in human serum sample *ex vivo*.

Time Point	Radiochemical Purity
15 min	99.5%
30 min	99.3%
60 min	99.0%
90 min	99.0%
120 min	98.4%
180 min	97.6%

## Data Availability

All the collected data for this manuscript was reported within the article.
